# Analysis of the Functional Morphology of Mouthparts of the Beetle *Priacma serrata*, and a Discussion of Possible Food Sources

**DOI:** 10.1673/031.013.12601

**Published:** 2013-11-12

**Authors:** Thomas Hörnschemeyer, Jake Bond, Philippe G. Young

**Affiliations:** 1 Georg-August-University Goettingen, Johann-Friedrich-Blumenbach-Institute for Zoology and Anthropology, Berliner Str. 28, 37073 Goettingen, Germany; 2 School of Engineering, Computing and Mathematics, University of Exeter, Harrison Building, North Park Road, Exeter, EX4 4QF, United Kingdom

**Keywords:** Coleoptera, Cupedidae, feeding, finite element analysis, high-resolution X-ray tomography, mandible mechanics, mandible stress, scanning electron microscopy

## Abstract

With the help of scanning electron microscopy, high resolution X-ray tomography (µCT), and finite element analysis, the mechanical and functional properties of the mandibles and associated muscles of the beetle *Priacma serrata* (LeConte) (Coleoptera: Archostemata) were studied. The combination of these techniques allowed for studying mechanical properties of the headmandible- system without using living animals. The µCT analysis delivered precise volumetric data of the geometry of the system to be studied. Dimensions of the cuticle of the parts involved could be readily deduced from the µCT-data. Thus, an exact representation of the specimen without significant artifacts like deformations and misalignments, usually resulting from histological sectioning, could be reconstructed. A virtual 3D model built from these data allowed for investigating different stress scenarios with finite element analysis. Combining these methods showed that *P. serrata* most likely uses its robustly-built mandibles for cutting hard material. In combination with available information on its habitat, possible food sources are discussed.

## Introduction

An important aspect for understanding nature and the interactions of organisms is to know the biological capabilities and requirements of as many species as possible. For example, knowledge of species' life cycles, modes of feeding, preferred habitats, etc., is crucial to understand how ecosystems are functioning. Unfortunately, this knowledge is still very scarce for the largest percentage of insect species.

The best way to learn about the biology of a species is to observe its actions in its natural environment; this, unfortunately, often is not possible, because many insect species are very rare and difficult to find or they have not yet been observed alive at all, and often only the type specimens that were used to describe it are known. Under these circumstances, a helpful, indirect way to deduce biological capabilities of a species is to investigate its functional morphology.

The beetle *Priacma serrata* (LeConte) (Coleoptera: Archostemata) is a characteristic example of a species whose biology is entirely unknown, although its morphology has been investigated in great detail (Edwards 1953a, b: male genitalia; Atkins 1958: wings and flight; Ross and Pothecary 1970: 1^st^ instar larva; Baehr 1975: thorax).

**Figure 1.**
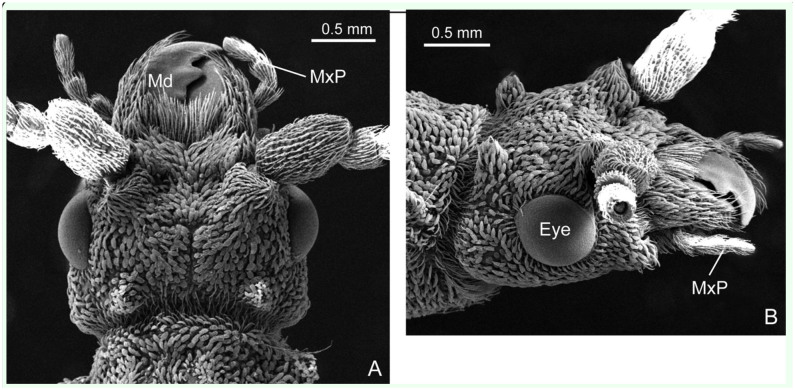
SEM images of the head of the beetle *Priacma serrata*. (A) Dorsal view. (B) Oblique right-lateral view. Md: mandible; MxP: maxillar palpus. High quality figures are available online.

The results of the study of the head morphology by Hörnschemeyer et al. (2002) are used as basis for this investigation. To analyze the mechanics of all mouthparts with finite element analysis (FEA) would be extremely time consuming and would require excessive computing power. So, the investigation was restricted to the mandibles, which are the largest elements (Figure 1) of the mouthparts and are equipped with the most massive head muscles. They also might be of prime importance for the animal to gain access to its food sources.

**Figure 2.**
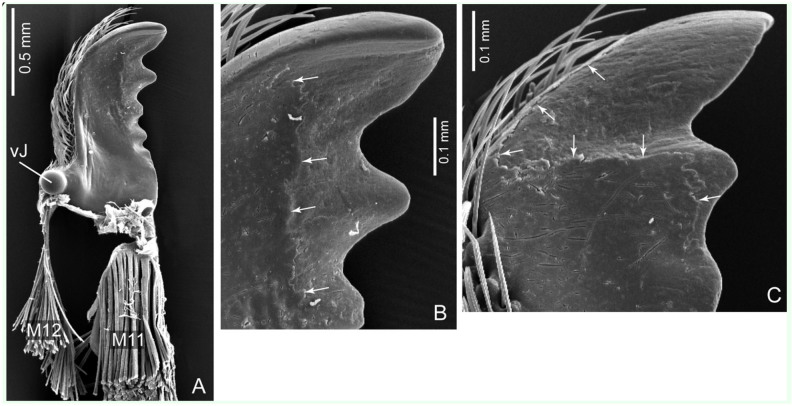
SEM images of mandibles of the beetle *Priacma serrata*. (A) Ventral view of right mandible. (B) Detail of tip, ventral view, right mandible. (C) Detail of tip, dorsal view, left mandible. M11, M12: abductor and adductor muscles; vJ: ventral joint. Arrows mark margin of eroded surface. High quality figures are available online.

**Figure 3.**
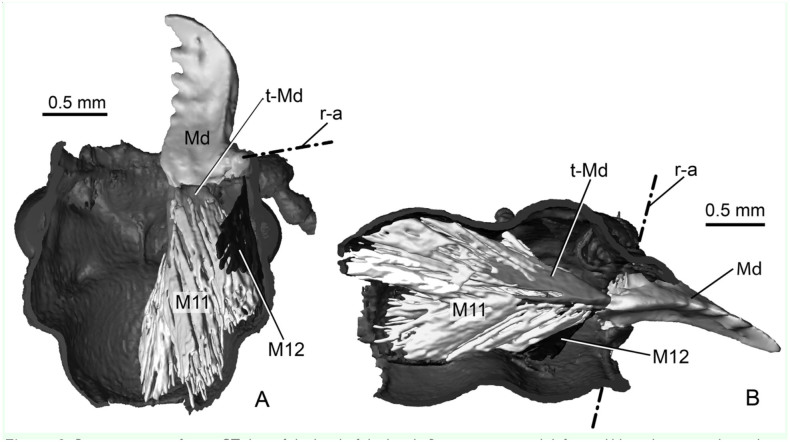
Reconstructions from µCT data of the head of the beetle *Priacma serrata* with left mandible and associated muscles. (A) Ventral view of dorsal half of head. (B) Right-lateral view of left half of head. M11: adductor muscle; M12: abductor muscles; Md: mandible; r-a: rotation axis of mandible; t-Md: tendon of M11. High quality figures are available online.

Each mandible is attached to the head capsule via two joints. The posterior or ventral joint is the older one in evolutionary terms. It is composed of a ball on the posterior outer rim of the mandible and a corresponding socket in the head capsule. The anterior or dorsal joint is built vice-versa, i.e., the ball is on the head capsule and the socket on the mandible (Figure 2, 2C). Two muscles, one adductor (closing, M11) and one abductor (opening, M12) (Figure 3) move each mandible. The adductor (M11) is by far the largest muscle in the head of *P. serrata* and also of many other insects. In *P. serrata*, its origin occupies nearly all available surface of the same (ipsilateral) half of the head. The insertion on the mandible is mediated by a broad tendon that is nearly as long as the mandible itself. The abductor (M12) is significantly smaller. It originates from the ventro-lateral anterior half of the head capsule and inserts via a narrow tendon on the outer rim of the mandible, between the dorsal and ventral joints.

The food source and the way of feeding of *P. serrata* are unknown. This lack of knowledge prevents studying this species in live experiments, even though it can be easily collected (Edwards 1951; Atkins 1957), because it is not possible to keep specimens alive in the laboratory.

To gain a better knowledge of the biology of *P. serrata* is highly desirable because it belongs to a very ancient beetle group, the Archostemata (Hörnschemeyer 2005, 2009). Fossils of this group have been found in sediments as old as 250 million years (lower Permian) (Beutel 1997; Beutel et al. 2007). Many of the extant archostematan species, including *P. serrata*, are morphologically very similar to these earliest fossils of Coleoptera. Any information that can be gained about the biology of the extant Archostemata will also increase the understanding of these ancient beetles and thus of the origin and evolution of the Coleoptera.

Combining scanning electron microscopy (SEM), high resolution X-ray tomography (µCT), and FEA allowed the mechanical and functional properties of the mandibles and associated muscles of *Priacma serrata* to be determined even though observations of living insects were not possible. These analyses could be done because the µCT dataset is an exact representation of the geometry and dimensions of the system to be studied. A virtual 3D model built from these data allowed investigating different stress scenarios with FEA.

Giving a detailed introduction into the basics of µCT and FEA here is far beyond the topic of this paper. However, a comprehensive account of µCT investigation of insects is give by Betz et al. (2007) and examples of the application of CT-data in such investigations are found in, for example, Beutel et al. (2007) or Hörnschemeyer et al. (2006). General information on FEA and its application in zoology can be found in Rayfield (2007).

A central aim of the virtual experiment was to learn how the mandibles of *P. serrata* and the associated muscles interact and how stress is distributed in the mandibles. Additionally, possible points of weakness in the system of mandibles, head capsule, and associated muscles were assessed. Behind these details stands the question on whether *P. serrata* uses its mandibles to process and/or access food or whether they are “only” there to impress other males in competitions for females as, for example, in the European stag beetle, *Lucanus cervus*. In combination with data available on its environment and behavior, possible food sources of *P. serrata* are discussed.

## Materials and Methods

### Specimens

All 12 specimens used for the investigation were collected by T. Hörnschemeyer in the vicinity of Bozeman, Montana, USA, in June 1996. The specimens were fixed in Dubosq- Brasil (Romeis 1968) and transferred to 80% ethanol after two days.

### Methods

For SEM investigation, the mandibles of 11 specimens were removed, dehydrated using absolute ethanol, dried at the critical point (Balzers CPD30 Critical Point Drier, Leica, www.leica-microsystems.com), sputter-coated with gold (Balzers SCD050 Sputter Coater), and investigated in a Leo 438VP SEM (Carl Zeiss Microscopy, www.zeiss.com).

The head of the specimen for the µCT investigation had a width of 2.1 mm and a single mandible measuring approximately 0.6 × 1.3 mm. It was dehydrated via absolute ethanol and dried at the critical point. The dry specimen was mounted on a standard SEM specimen holder with dental wax. The measurement was made with a Skyscan 1072 highresolution µCT system at 80 kV and 100 µA (http://www.skyscan.be/home.htm). The resulting dataset comprised 356 2D slices with a voxel size of 5.429 × 5.429 × 10.858 µm. Visualization of the resulting data was partly done with Amira 4.0 (Visage Imaging Inc., www.amira.com).

The 2D slices from the µCT data were combined into a 3D model using the program ScanIP (Simpleware Ltd., http://www.simpleware.com/). The identification of cuticle and the differentiation between mandible and head capsule were done with a combination of automatic recognition of grey value distribution with subsequent manual correction. In order to remove errors within the preliminary 3D model, a floodfill operation was used to eliminate specified unconnected regions. The antennae of the specimen were manually removed from the model in order to reduce the number of elements present in the analysis. The voxel model was imported into +ScanFE (Simpleware Ltd., www.simpleware.com) and smoothed with the following parameters: extra surface smoothing; curvature cutoff at 0.5; maximum iterations of 2; and a minimum quality target of 0.15. All other parameters remained at the default values.

**Figure 4.**
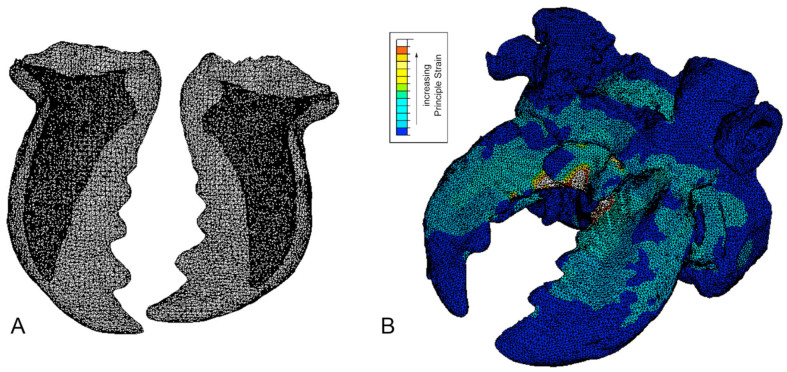
(A) Tetrahedral mesh of mandibles of *Pricama serrata*, ventral view. Dark grey areas represent hollow sections of the mandibles. (B) Tetrahedral mesh of mandibles and anterior part of head with exemplary rendering of principal strain. High quality figures are available online.

To prepare the data for the FEA with the program Abaqus (Dassault Systémes, http://www.3ds.com/), the model was divided into two separate material types: the mandibles and the rest of the head structure. To reduce the complexity of the model so that analysis was possible with the available computing power within a reasonable timeframe, the original data were resampled so that the resulting mesh consisted of fewer elements at the expense of model accuracy. A cubic resampling was done to a pixel spacing of 0.02169 mm (resample 50%), arriving at a tetrahedral mesh with node and element numbers as given in Table 1. Comparison with SEM images (Figure 2) showed that the resulting model accurately reproduced the topology of the head and mandibles, including the hollow interior of the mandibles (Figure 4A).

**Table 1.**
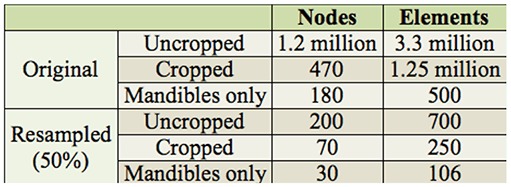
Number of nodes and elements of mesh.

**Table 2.**
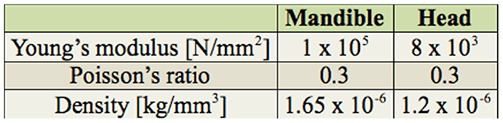
Material input parameters.

The input requirements when specifying an elastic material in Abaqus are density, Young's modulus, and Poisson's ratio. Obtaining material data specific to *P. serrata* was not possible, but comparisons with other insect species can be drawn. Flannigan (1998) suggests a Poisson's ratio for insect cuticle of 0.3. The Poisson's ratio for numerous materials falls within a narrow band between 0.25 and 0.3 (Hibbler 1993), which suggests that a value of 0.3 is a suitable material property. Values for density and Young's modulus of insect cuticle were taken from Vincent et al. (2004). The material properties of chitin vary greatly depending on its implementation. The head cuticle can be likened to that of the elytron, which is considerably less hard than the mandibles, particularly the cutting face of the mandibles. However, there are no specific data for the required parameters for the mandibles, but a comparative estimate can be made using the available data. A study into the hardness of locust mandibles showed that the cutting face was almost twice as hard as the rest of the mandible (Hillerton et al. 1982). The material parameters used in the model beetle's mandibles are shown in Table 2.

To best determine the functional morphology of the beetle mandibles, a number of load states were applied, both for the mandibles alone and for the mandible with head attachment (Figure 4B). For each case the boundary conditions were adjusted such that meaningful results could be expected while keeping the complexity of the model within the available computing power.

**Table 3.**
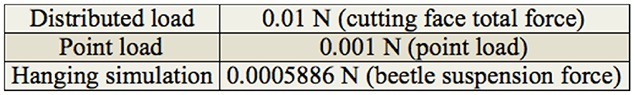
Load states.

**Figure 5.**
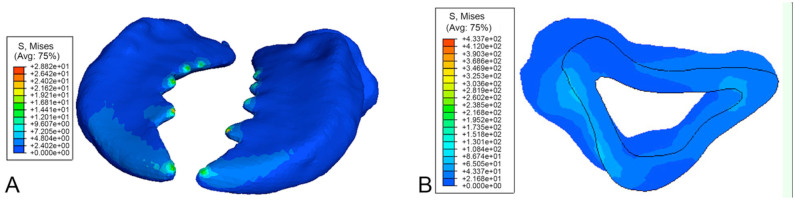
Mandibles of *Priacma serrata* with von Mises stress contours for point load case. (A) Dorsal view. (B) Cross-section of left mandible. High quality figures are available online.

**Figure 6.**
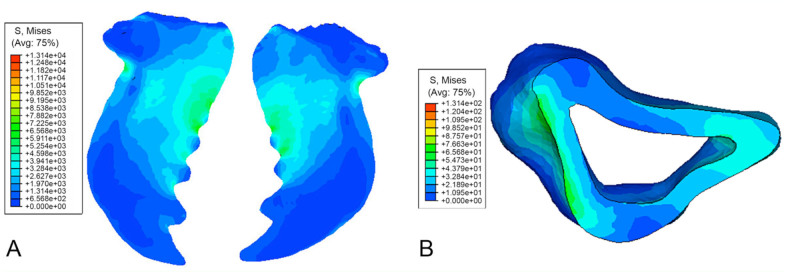
Mandibles of *Priacma serrata* with von Mises stress contours for distributed load case. (A) Ventral view. (B) Crosssection (both after Bond et al. 2008). High quality figures are available online.

**Figure 7.**
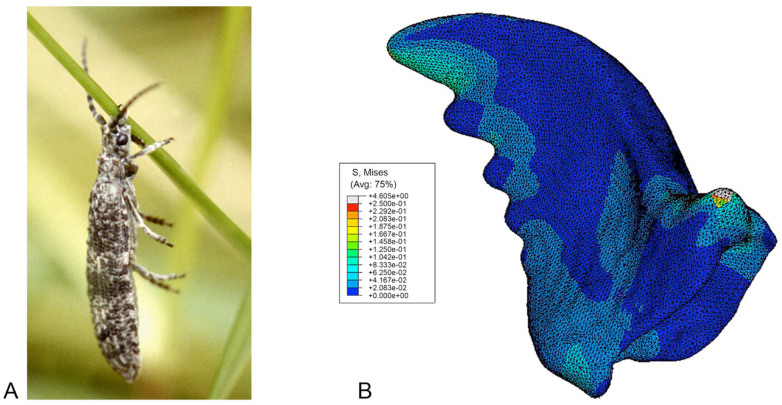
(A) *Priacma serrata* clinging to a blade of grass before being collected, Montana, USA, June 1996. Example for the third load state, see Materials and Methods (after Bond et al. 2008). (B) Right mandible in ventral view with von Mises stress contours for hanging case. High quality figures are available online.

For the initial point load state, point loads were applied to each of the four mandible peaks perpendicular to the longitudinal axis of the mandible (Figure 5). Constraints were imposed on the rear of the mandible to the effect that elements were prevented from translational or rotational movements by applying a fixed displacement of 0. This resulted in the mandibles being locked in position and loaded such that the mandibles were directed away from the central body line. Similarly, for the body model the mandibles were loaded in the same manner, and the constraints were imposed on the rear face of the head such that the load was transmitted to the interface between mandible and head. The loads on the mandibles were altered to simulate different load states. A uniform load along the cutting edge of the mandibles simulated the clasping of an object over the entire cutting edges (Figure 6). The third load state simulated the beetle gripping onto a leaf, as illustrated in Figure 7. In order to simulate the weight of the beetle, loads were applied on the proximal faces of the teeth. It can be assumed that the teeth were fully embedded into the grass leaf from which they hung such that the weight was acting distally. The weight of the specimen was approximated to 0.06 g (calculated as the mean value from a measurement of ten specimens from the same population as the specimen investigated), which was then divided equally among the mandible teeth. Table 3 summarizes the loads exerted upon the beetle mandibles in each load state.

### Glossary of FEA terms used in the text

Poisson's ratio: For objects that are stretched (or compressed), Poisson's ratio is the ratio of the contraction perpendicular to the applied load to the extension in the direction of the applied load. In the case of compression, it is the ratio of extension perpendicular to the load to compression in the direction of the load. Values for most materials are in the range of 0 to 0.5 (Ragab and Bayoumi 1999).

Principal strain: describes the deformation of an object in principal directions given as the amount of length change in relation to the original length (Ragab and Bayoumi 1999).

Total strain energy: describes the work that is utilized when deforming a structure (Dumont et al. 2009).

von Mises stress: Stress is a tensor quantity with nine components. The von Mises stress is a stress index especially suited for failure analysis and is a combination of these components (Ragab and Bayoumi 1999).

Young's modulus: With the help of Young's modulus it can be calculated how much the length of an object changes under compression or under tension. It describes the elasticity of the material in units of pressure. Here, N/mm^2^ is used (Ragab and Bayoumi 1999).

## Results

### Finite element analysis

In the following sections, stress is always given as von Mises stress. Studying von Mises stress, rather than each of the nine components of stress, allows for meaningful interpretation of the results (Ragab and Bayoumi 1999; Raghavan et al. 2000). Other parameters of stress or strain (e.g., total strain energy (Dumont et al. 2009)) closely replicated the results for Mises stress; therefore, in the following only the results for one parameter are shown.

The von Mises stress for a distributed load upon the mandibles is shown in Figure 6. The load was distributed upon both mandibles' cutting edges from the tip of the mandibles extending to the fifth tooth. A force of 0.001 N was applied to the nodes on the inner face of the mandible, which approximated to 0.01 N distributed force and simulates a likely failure scenario under which the cuticle might yield.

The peak stress was experienced in the area behind the ridged teeth on each mandible. There were also increased stresses experienced at the outer edge of the mandible, where the material was under compression (Figure 6B). Thus, it is probable that failure will occur proximally along the mesal cutting edge in each mandible. There was minimal stress experienced by the thicker distal section of the mandible.

In the point load scenario, the point loads on the mandible cutting edge (Figure 5A) were considerably smaller than the distributed loads. The contours showed that there were still compressive forces acting on the outside of the mandible and tension at the filleted edge of the cavity closest to the cutting edge.

In the third scenario, the distally oriented loads at the peaks of the mandible cutting edge simulated the weight of the beetle acting against the clasping of the beetle to a grass leaf (Figure 7A). In contrast to the other load states, the stress concentrations were found at the distal edge of the mandibles, with the proximal edge experiencing comparatively less stress (Figure 7B).

### Scanning electron microscopy

The mandibles investigated showed a characteristic and remarkably steady pattern of abrasion (Figure 2B, C). The most conspicuous wear was present in the distal part of each mandible, usually back to the third tip. In 10 of 11 pairs of mandibles investigated, the defects were most severe on the ventral surface of the left mandible and on the dorsal surface of the right mandible. In one pair, the pattern was inversed, with the dorsal surface of the left and the ventral surface of the right mandible showing the strongest abrasion. This corresponded to the observation (T. Hörnschemeyer, unpublished data) that in most specimens of *P. serrata* the left mandible is positioned above the right one when the mandibles are closed. Only very few specimens show the inverted arrangement.

In addition to the loss of a thin layer of cuticle over more or less expanded areas next to the cutting edge, as seen in Figure 2, there were also deep scratches in the otherwise intact surface. These scratches were of variable length and density and they were oriented mostly perpendicular to the generalized direction of the cutting edge. Like the abrasion, the scratches also were restricted to the anterior half of the mandible.

## Discussion

In this investigation, information on the biology, i.e., the feeding habit and the food source, of *P. serrata* was deduced from morphological data and from the simulation of physical properties through the combination of µCT and FEA supplemented with SEM images. The application of FEA on data produced with µCT was novel, at least in entomology. In this study, the comparatively high resolution of the µCT data turned out to be a major challenge for the analysis. The original finite element model generated from the µCT data turned out to be far too complex to be processed with the available computing power. Fortunately, reduction of the amount of data with the software tools available did not influence the representation of the structures of interest in a negative way. The fine structure of the somewhat irregularly sculptured outer surfaces of the mandibles and of the head was lost, but the substantial proportions and dimensions of these parts were preserved well enough so that the results were biologically and physically meaningful.

In the case of *P. serrata*, no observations of the kind of food that the adult feed on, or if they feed at all are available. An investigation of the intestines of several specimens did not produce any identifiable particles. In fact, they were nearly completely empty. This, however, may be attributable to the collecting conditions. The animals had to be kept alive for some time after collection, probably without an adequate food source, and therefore they had no possibility to feed during this time (personal observation T. Hörnschemeyer).

The mandibles look quite strong, but in insects this does not necessarily mean that they are used for feeding. They could also be used as a kind of weapon or just for show when males compete for resources like females or territories, as in some stag beetles (Lucanidae).

Nevertheless, the finite element analysis based on µCT data combined with SEM investigation of the mandible surfaces showed that the mandibles were built to withstand comparatively strong forces. The possible points of failure of the mandible structure were the mesal cutting edge posterior of the teeth and the narrow point of attachment of the tendon of the adductor muscle (M11; Figure 2A). The stress was most intense at the cutting edge, and the structures seem to be weakest compared to the occurring stress at the attachment point of the tendon. However, under natural conditions it seems to be extremely unlikely that failure at the tendon attachment will ever occur.

This investigation illustrates that it is possible to simulate complex problems with a high degree of accuracy by combining µCT and image-based meshing techniques. FEA simulations of a model based on µCT data can be very helpful to understand the functional morphology of certain parts of an insect's body, especially where observations of living specimens are not possible.

The additional SEM examination of the surfaces of the mandibles showed that there was heavy abrasion in certain areas, which indicates that the mandibles are used for cutting comparatively tough materials rather than just for impressing male competitors.

The kind of abrasion observed in the mandibles also makes threatening behavior, like rubbing the mandibles together to produce sound for repelling possible predators or competitors (as other beetles are capable to do (Drosopoulos and Claridge 2006)), seem unlikely. For one, there were no structures present that would support the generation of sound, and secondly the abrasions were far too irregular as to be produced by rubbing the mandibles against each other. Also, there was significant abrasion where the mandibles never touch, e.g., on the ventral side of the ventral mandible and *vice versa*. Furthermore, there always seemed to be some free space between the two mandibles, even when they were completely closed.

Another possibility for producing the abrasions might be when a beetle gnaws itself out of the pupal chamber where its metamorphosis took place. For other cupedid beetles (e.g. *Tenomerga* (Fukuda 1938, 1941), it is known that their larvae develop in wood, and the pupal chamber is gnawed into the wood directly under the bark by the last larval instar. However, for *P. serrata* it is not known where larvae develop or where the pupa rests. If gnawing through wood or bark would cause the abrasions on their mandibles, given their design one would expect a wear pattern where the heaviest abrasions should be present in the anterior most part of each mandible. Especially the front-most surfaces and the first teeth would suffer the most. However, this is not what can be observed. The strongest abrasions are usually present in the area from the second to the last tooth of each mandible, suggesting that whatever they cut is more or less freely accessible and they do not have to dig into it with the first teeth. Additionally, the shape of the mandibles does not support the assumption of the freshly hatched beetle gnawing its way out of a wooden pupal chamber. The proportions of the levers in the mandibles are very inadequate for such a usage, as at the tips of the mandibles the beetles cannot exert much force. In beetles that have to gnaw their way through tough material, the mandibles usually are quite short (less than half as long as in *P. serrata*, in relation to the length of the head) and often have a more vertically oriented teethed cutting edge, as for example in *Tenomerga* (Fukuda 1939; Neboiss 1968) or in Buprestidae (Böving and Craighead 1931; Marannino and de Lillo 2007; Chamorro et al. 2012).

What the beetles chew is still unknown. It is obvious that *P. serrata* uses its mandibles to cut tough material. *P. serrata* occurs throughout the Rocky Mountains (western North America), usually in the vicinity of Douglas Fir, *Pseudotsuga menziesii*, forests. If the beetle feeds on this plant, which seems to be very likely, there remain two possible food sources. The beetles might use their strong mandibles to cut into the bark and wood, probably of smaller twigs, to get to the sap of the tree, or they might open the fir cones to feed on the seeds. Since *P. serrata* does not have mouthparts specialized for taking up fluids (Hörnschemeyer et al. 2002), feeding on seeds may be the more plausible possibility. Nevertheless, this interpretation certainly needs corroboration through observations in the field.

Even though the combination of FEA and µCT can already produce very valuable results, their application in entomology can be further improved. At present, precise data for the material properties of the cuticle in different areas of the body of an insect are not readily available. Measurements of the cuticle parameters of different body parts and for representatives of various groups of insects would produce the base for more complex and still more precise FEA of mechanical properties of the insect body.

Such FEA simulations could lead to a much better understanding of a wide range of insect behavior, including food processing as in the present investigation. More detailed knowledge of material properties would allow for much more detailed analyses. Other areas might even be more interesting, such as the jumping mechanisms in click beetles (Elateridae; Ribak and Weihs (2011)) or in Collembolans (Brackenbury and Hunt 1993). Results of such analyses might be interesting for engineering purposes.

## Abbreviations:

**FEA**,: finite element analysis;
**SEM**,: scanning electron microscopy;
**µCT**,: high resolution X-ray tomography
